# Methyl viologen-templated zinc gallophosphate zeolitic material with dual photo-/thermochromism and tuneable photovoltaic activity†
†Electronic supplementary information (ESI) available: Crystal data and structure refinement; thermal ellipsoids; 3D channel system, tiling structure, LC-HRMS, solid-state ^13^C NMR, liquid ^13^C NMR, PXRD, TG, EPR. CCDC 1008498. For ESI and crystallographic data in CIF or other electronic format see DOI: 10.1039/c5sc00291e



**DOI:** 10.1039/c5sc00291e

**Published:** 2015-03-05

**Authors:** Junbiao Wu, Chunyao Tao, Yi Li, Jiyang Li, Jihong Yu

**Affiliations:** a State Key Laboratory of Inorganic Synthesis and Preparative Chemistry , College of Chemistry , Jilin University , Changchun 130012 , P. R. China . Email: lijiyang@jlu.edu.cn ; Email: jihong@jlu.edu.cn ; Fax: +86 431 8516 8608 ; Tel: +86 431 8516 8608

## Abstract

A novel zinc gallophosphate zeolitic material |(C_12_H_14_N_2_)_4_F_1.33_|[Ga_13.33_Zn_6.67_(PO_4_)_20_] (denoted as JU101) has been prepared by using *in situ* generated methyl viologen (MV) as the template. The framework of JU101 features two building units including an unprecedented fused d6r and a novel [4^12^·6^4^·8^2^·10^2^] cavity. The connection of these two building units forms a 3D intersecting pore system containing 8-rings along the [010] direction, and 10-rings along the [001] and [100] directions. The MV-templated JU101 zeolitic material offers a new type of electron transfer system, which endows the material with interesting photochromism, thermochromism, and tuneable photovoltaic activity in response to light and heating. Importantly, JU101 shows an extended photochromism range from UV to visible light, high thermal stability, as well as a long-lived charge-separated state for potential application in solar energy conversion.

## Abstract 2

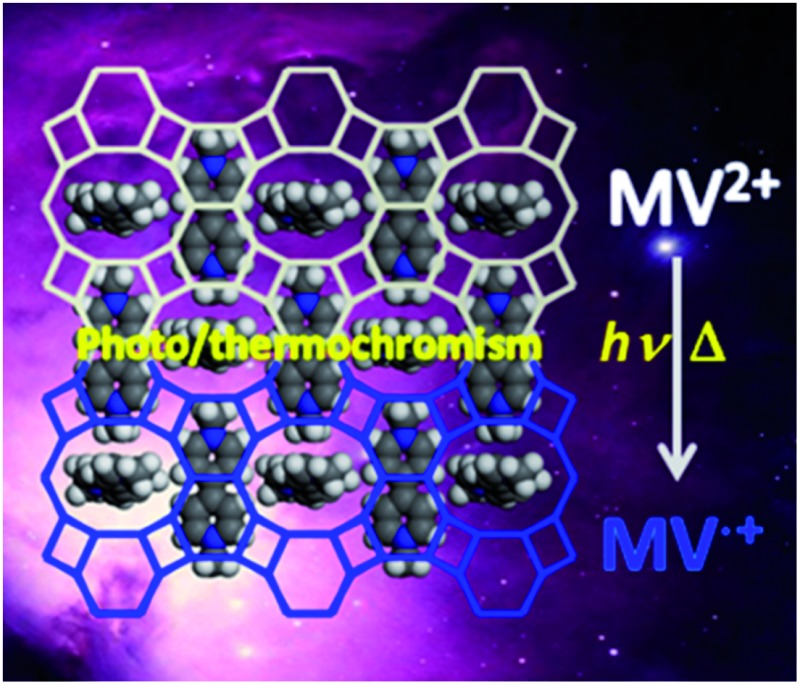
The first zeolitic material templated by MV^2+^ cations exhibits dual photo-/thermochromism with ultralong-lived charge separation and high thermal stability, as well as tuneable photovoltaic activity.

## Introduction

Zeolites are an interesting class of inorganic crystalline microporous materials constructed by corner-sharing TO_4_ (T = Si, Al, P, *etc.*) tetrahedra.^1^ These materials have wide applications as catalysts, adsorbents, and ion-exchangers.^2^ In addition, zeolitic materials have continued to find new applications in ion and proton conduction, medicine, microelectronics, *etc.* ^3^ The discovery of new zeolites with unique structure characteristics and good properties is of both academic and industrial relevance.

In recent years, important advances have been achieved in the synthesis of new zeolite structures by using novel diquaternary ammonium cations as organic templates or structure-directing agents (SDAs).^4^ Notable examples are a number of silicate and germanate zeolites, such as SSZ-74 (-SVR), IM-5 (IMF), TNU-9 (TUN), ITQ-37 (-ITV), ITQ-39 (*-ITN), *etc.* ^5–9^ These diquaternary SDAs typically possess complex structures of rigid rings and flexible chains. Notably, methyl viologen cation (MV^2+^) is a diquaternary ammonium cation, but it has not been used in zeolite synthesis yet. More interestingly, MV is photochromic because of the photocatalyzed electron transfer from the counter-anion to the MV^2+^ cation which promises MV-containing materials potential applications in photonic devices, X-ray detectors, and oxygen detectors.^10–12^ Indeed, MV as an efficient electron-transfer media has been doped in several zeolites including NaX, NaY, Naβ, Na-MOR, zeolite L, and NaZSM-5 by ion exchange to achieve long-lived charge separation for solar energy conversion.^13–17^ The rigid microporous zeolite matrix can stabilize the unstable radical cations and increase their lifetimes. However, only a limited amount of MV^2+^ can be incorporated into zeolites and the dealkylation/decomposition of viologens could occur during ion exchange.^18^

So far, several photochromic materials based on metal–organic frameworks (MOFs), inorganic–organic hybrid structures, and inorganic open frameworks have been prepared by using MV as ligands or SDAs.^19–25^ Studies show that the photochromic behaviours of MV are strongly influenced by the electron-transfer pathway between the surrounding anionic framework and MV^2+^ cations. We expect that novel zeolite structures may be produced by using MV as the template; meanwhile, the resulting MV-templated zeolites may function as a new efficient electron-transfer system for potential applications in photochromism and solar energy conversion.

Here, we present the first example of zeolitic material synthesized by using MV^2+^ cations as the SDA, which exhibits interesting dual photochromism and thermochromism, as well as tuneable photovoltaic activity in response to light and heating. The new zinc gallophosphate zeolitic material |(C_12_H_14_N_2_)_4_F_1.33_|[Ga_13.33_Zn_6.67_(PO_4_)_20_] (denoted as JU101) has a 3D intersecting channel system of 8-rings and 10-rings. Significantly, JU101 shows a wide spectral range of photochromism from UV to visible light and an ultralong-lived charge-separated state.

## Experimental section

### Materials

The source materials used in a typical synthesis of JU101 were zinc acetate dihydrate (Zn(OAc)_2_·2H_2_O, 99.0%, Tianjin Fuchen Chemical Reagents Factory); gallium(iii) oxide (Ga_2_O_3_, 99.999%, Sinopharm Chemical Reagent Co., Ltd); phosphorous acid (H_3_PO_3_, 99.0%, Sinopharm Chemical Reagent Co., Ltd); 4,4′-bipyridine (C_10_H_8_N_2_·2H_2_O, 98.0%, Shanghai Kefeng Industry & Commerce Co., Ltd) and methanol anhydrous (CH_3_OH, 99.5% Tianjing Guangfu Fine Chemical Research Institute). The reagents and solvents employed were used as received without further purification.

### Synthesis of JU101

JU101 was obtained from a reaction mixture of Zn(OAc)_2_·2H_2_O (0.22 g, 1 mmol), Ga_2_O_3_ (0.375 g, 2 mmol), H_3_PO_3_ (0.328 g, 4 mmol), 4,4′-bipyridine (0.384 g, 2 mmol) and HF (0.1 ml, 2.25 mmol) in a mixture solvent of methanol (5 ml) and water (2 ml) with a molar composition of 1.0 ZnO : 2.0 GaO : 2.0 H_3_PO_3_ : 2.0 4,4′-bipyridine : 2.25 HF : 124.0 CH_3_OH : 117.0 H_2_O at 170 °C for 7 days in a Teflon-lined stainless steel autoclave. The colourless crystals of JU101 mixed with an amorphous phase were filtered and washed with distilled water, and then dried at room temperature. The crystals of JU101 were selected for further characterization and property study. The MV^2+^ cations were *in situ* generated by the reaction of 4,4′-bipyridine and methanol, which is less toxic and more efficient compared to the direct use of MV. The formation of MV^2+^ cations was confirmed by liquid chromatography-high resolution mass spectrometry and solid-state ^13^C NMR (Fig. S1 and S2, ESI†). The initially added H_3_PO_3_ was oxidized into PO_4_^3–^ anions in this reaction system. The phase purity of selected crystals was confirmed by the good agreement between the experimental XRD pattern and the simulated one based on structure analysis (Fig. S3, ESI†).

### Characterizations

Powder X-ray diffraction (PXRD) and temperature dependent powder X-ray diffraction data were collected on a Rigaku D/max-2550 diffractometer with Cu K_α_ radiation (*λ* = 1.5418 Å). Temperature dependent powder X-ray diffraction data were collected from room temperature to 600 °C with a heating rate of 10 °C min^–1^. Inductively coupled plasma (ICP) analysis was performed on a Perkin-Elmer Optima 3300DV spectrometer. Elemental analysis was conducted on a Perkin-Elmer 2400 elemental analyzer, found C 12.88%, H 1.64% and N 2.57%. F^–^ ion selective electrode analysis was performed on a Mettler Toledo instrument to confirm the existence of F^–^ ion in the compound. Thermogravimetric (TG) analysis was carried out on a TA Q500 analyzer in air with a heating rate of 10 °C min^–1^ from RT to 1200 °C. It gave an obvious weight loss of *ca.* 19.51 wt% from 400 to 1200 °C (Fig. S4, ESI†), corresponding to the loss of four organic MV^2+^ cations and 1.33 F^–^ ions in one unit cell (calcd 19.09 wt%). Liquid chromatography-high resolution mass spectrometry (LC-HRMS) was performed on a HPLC: Agilent 1290 and HRMS: MicroTOF-Q II (Bruker Daltonics, Bremen, Germany). ^13^C solid-state MAS NMR was performed at room temperature on an InfinityPlus-400 spectrometer operating at *B*_0_ = 9.7 T. Liquid ^13^C NMR spectrum was recorded using a Varian Mercuryvx 300 spectrometer. The UV/Vis absorption spectra were recorded at room temperature on a Shimadzu UV-2450 spectrophotometer. The electron paramagnetic resonance (EPR) spectroscopy was performed on a JEOL JES-FA200 EPR spectrometer. A 500 W high-pressure mercury lamp was used as an irradiation light source for *in situ* EPR measurements. The surface photovoltage (SPV) measurement system was composed of a monochromatic light source, a lock-in amplifier (SR830-DSP) with a light chopper (SR540), a sample cell and a computer. A 500 W xenon lamp and a double-prism monochromator provided the monochromatic light. In the photovoltaic cell, the powder sheet was directly sandwiched between two blank indium tin oxide (ITO) electrodes. The field-induced surface photovoltage spectroscopy (FISPS) was a supplement to the SPV spectroscopy method. In FISPS, the external electric fields were applied between the two electrodes. Transient state surface photovoltage (TPV) measurements for samples were collected by the process that the sample chamber connected an ITO glass as the top electrode and a steel substrate as the bottom electrode, and a 10 μm thick mica spacer was placed between the ITO glass and the sample to decrease the space charge region at the ITO–sample interface. The samples were excited by a radiation pulse of 355 nm with 10 ns width from the second harmonic of a neodymium-doped yttrium aluminum garnet (Nd:YAG) laser (Lab-130-10H, Newport, Co.). Intensity of the pulse was measured by a high-energy pyroelectric sensor (PE50BF-DIF-C, Ophir Photonics Group). The signals were amplified with a preamplifier and then registered by a 1 GHz digital phosphoroscilloscope (DPO 4104B, Tektronix). The TS-TPV measurements were performed in air atmosphere and at room temperature.

### Single-crystal structure determination

A suitable single crystal of JU101 with dimensions of 0.22 × 0.21 × 0.18 mm^3^ was selected for single-crystal X-ray diffraction analyses. The intensity data were collected on a Bruker SMART APEXII CCD diffractometer by oscillation scans using graphite-monochromated Mo Kα radiation (*λ* = 0.71073 Å) at a temperature of 23 ± 2 °C. Cell refinement and data reduction were accomplished with the SAINT processing program.^26^ The structure was solved in the space group *C*2/*m* (no. 12) by direct methods and refined by full matrix least-squares technique with the SHELXTL crystallographic software package.^27^ The heaviest atoms of Zn/Ga, P and O can be unambiguously located, and the C, N and F atoms were not located due to the high disorder. Therefore, the SQUEEZE routine of PLATON was used to remove the diffraction contribution from guests to produce a set of guest-free diffraction intensities.^28,29^ The existence of guest species in JU101, such as MV^2+^ cations and F^–^ ions, will be determined by compositional and TGA analyses. All non-hydrogen atoms were refined anisotropically. Experimental details for the structure determination are presented in Table S1, ESI.†

### Simulation method

Computer simulation was used to determine the positions of MV^2+^ cations in the pores of JU101. Calculations were performed in Materials Studio Software 4.0 using the Universal force field.^30^ The crystal symmetry of JU101 was decreased to *P*1 to fit the location of MV^2+^ cations. Based on the compositional and TG analyses, four MV^2+^ cations were added randomly into the pores of JU101 in one unit cell, and their positions were optimized by using energy minimization by fixing the inorganic framework. Such process was repeated several times to obtain more precise results, and was completed when the difference of the last two calculated energies was lower than 1 kcal mol^–1^.

## Results and discussion

### Structure of JU101

JU101 crystallizes in the monoclinic space group *C*2/*m* (no. 12). Its framework is built from TO_4_ (T = Ga, Zn and P) tetrahedra forming the anionic [Ga_13.3_Zn_6.67_(PO_4_)_20_]^6.67–^ framework, and the negative charges are compensated by disordered MV^2+^ cations and F^–^ ions occluded in the pores. The asymmetric unit contains one unique Ga site, two unique M sites (M = Ga/Zn), and three unique P sites (Fig. S5, ESI†). The unique Ga site is suggested by the typical Ga–O distances in the range of 1.781(5) to 1.796(6) Å, while the other two metal sites cannot be distinguished through X-ray diffraction because the average M–O bond length of 1.85 Å lies between the ideal Ga–O (1.82 Å) and Zn–O (1.93 Å) bond lengths. The ratio of Ga : Zn is approximately 2 : 1 on the basis of elemental analysis.

**Fig. 1:**
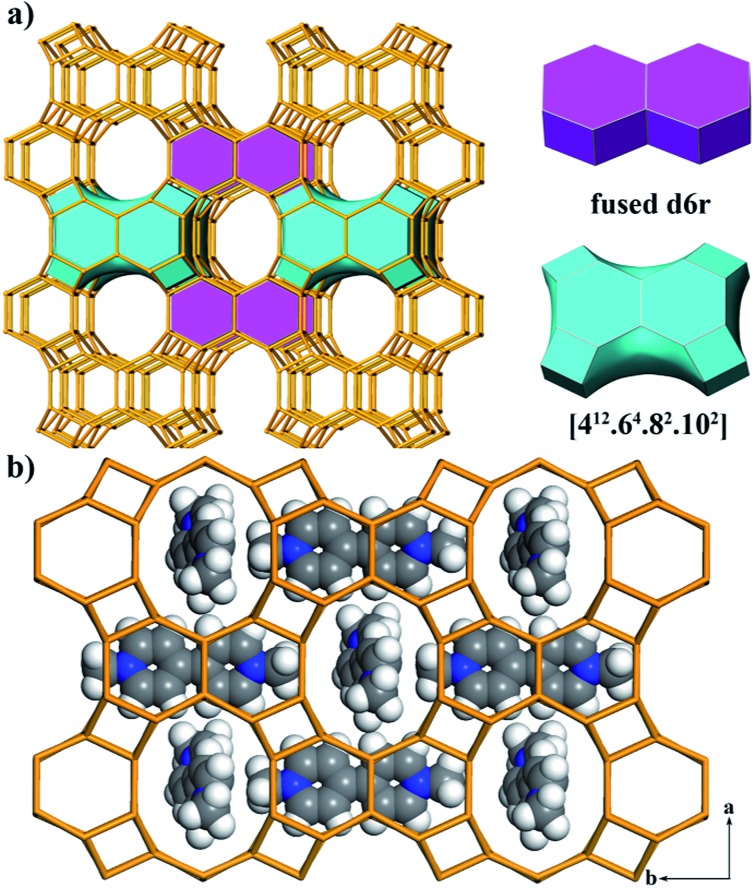
(a) 3D framework of JU101 showing 10-ring channels along the [001] direction (left). Two characteristic BUs, fused d6r and the [4^12^·6^4^·8^2^·10^2^] cavity (right) are highlighted in the framework. (b) Simulated positions of MV^2+^ cations in the pores viewed along the [001] direction. Colour: C, gray; H, white; N, blue.

The framework of JU101 features two characteristic building units (BUs): a fused d6r and a novel [4^12^·6^4^·8^2^·10^2^] cavity (Fig. 1a). Note that d6r is a basic BU for zeolite structure, but the fused d6r has never been found in previous known structures. The two types of BUs are linked alternately to form a 3D channel system (Fig. S6, ESI†). It possesses 10-ring channels running along the [001] and [100] directions, intersecting with 8-ring channels running along the [010] direction. So far, the intersecting 10-ring and 8-ring channels are only found in several aluminosilicate and germanosilicate zeolites.^31^ Such channel system is the first observed in phosphate-based zeolitic material. The framework density of JU101 is 15.3 T/1000 Å^3^.

The structure of JU101 can also be described in terms of natural tiling.^32^ The three-periodic net is carried by a unique natural tiling with a transitivity of (6 11 10 4). The signature of this tiling is [4^2^·10^4^] + 2[4^6^·6^2^] + [4^4^·8^2^·10^2^] + [4^12^·6^4^·8^2^·10^2^] (Fig. S7, ESI†). Four disordered MV^2+^ cations and 1.33 F^–^ ions are located in one unit cell based on the compositional and TG analyses, as well as the charge balance. The positions of MV^2+^ cations occluded in JU101 are theoretically simulated by using Materials Studio Software (Fig. 1b).^30^

### Photochromism and thermochromism

**Fig. 2:**
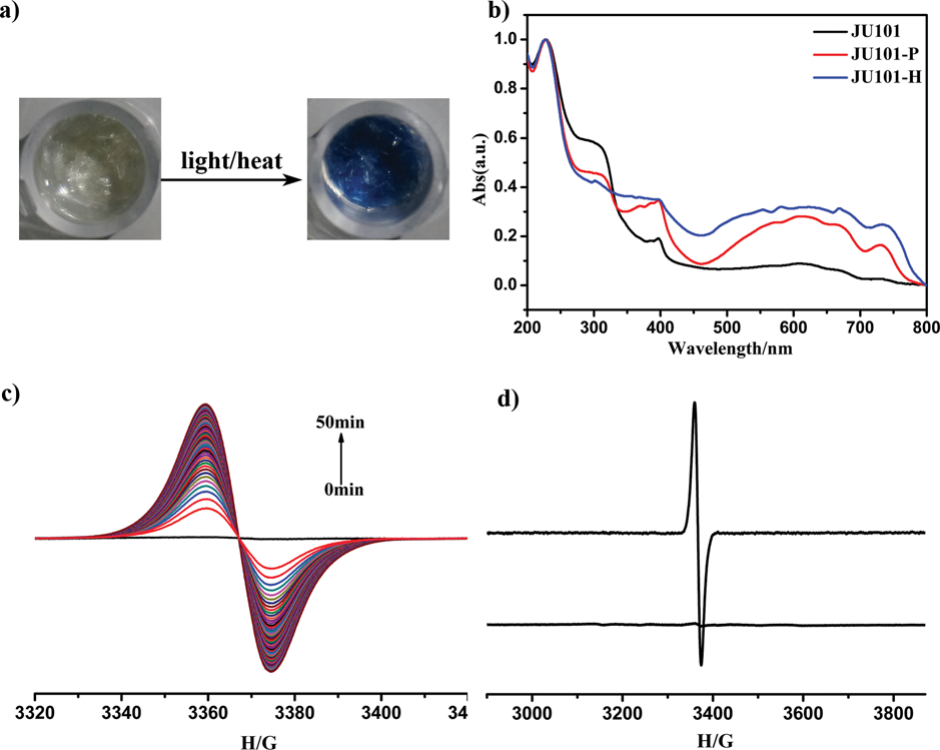
(a) Photo-/thermochromic phenomenon of JU101. (b) UV/Vis spectra of JU101, photo irradiated sample JU101-P, and heated sample JU101-H. (c) *In situ* time dependent EPR spectra of JU101 by UV irradiation. (d) EPR spectra of JU101 and JU101-H.

JU101 is the first MV-templated zeolitic material, in which the MV^2+^ amount is much higher than that in ion-exchanged MV-zeolites.^13–17^ As expected, JU101 is photoactive and undergoes a rapid visible photochromic transformation from colourless to blue upon light irradiation (20 W UV light for 5 min or sunlight for 10 min) at room temperature (Fig. 2a). As shown in Fig. 2b, a new absorption band at 620 nm is observed in the UV/Vis spectra for the photo irradiated sample (denoted as JU101-P), which is ascribed to the characteristic absorption of MV˙^+^ radicals reduced from MV^2+^ cations *via* electron transfer.^18–25^ *In situ* time dependent electron paramagnetic resonance (EPR) measurement at room temperature shows that no EPR signal is detectable for the original sample, but a strong signal at *g* = 2.0031 is observed after irradiation corresponding to the viologen free radicals (Fig. 2c).^19–25^ This indicates that MV^2+^ cations are reduced to MV˙^+^ radicals after photo irradiation, suggesting that the electron transfer occurs between the anionic zeolite framework and the MV^2+^ cations. Similar electron transfer phenomenon has also been observed in the reported MV-based MOFs, inorganic–organic hybrid structures, and inorganic open frameworks.^19–25^ The MV˙^+^ radicals are generated rapidly upon UV irradiation for 1 min, and the intensity of the EPR signal increases with prolonged irradiation time, finally tending to saturation (Fig. S8, ESI†). Accordingly, the colour of the irradiated samples gradually changes to deeper blue.

Extending photoinduced energy to the visible spectrum is of significance for the utilization of solar energy.^33^ Interestingly, the photochromic behaviour of JU101 is also observed under visible light illumination (*λ* > 420 nm, about 1 h) (Fig. S9, ESI†). This suggests that JU101 requires lower threshold energy for photochromism than the UV-induced photochromic zinc phosphate JU98 and X-ray-induced photochromic bimetal phosphate JU99 that both contain MV^2+^ cations.^24,25^ The shortest electron-transfer pathway between the MV^2+^ acceptor and the anionic-framework donor in JU101 is 3.28 Å (N···O) on the basis of structure simulation, which is much shorter than that of JU99 (4.52 Å) and JU98 (3.45 Å). This may explain why JU101 is also sensitive to visible light. The different photochromic behaviours exhibited by these three compounds demonstrate that photoinduced colour change of MV^2+^ is strongly influenced by the surrounding inorganic anionic framework.

In general, the photochromic transformation can be recovered by heating as in the cases of JU98 and JU99. However, no noticeable colour change is observed upon heating JU101-P at 230 °C for 24 hours. Strikingly, its blue colour becomes deeper upon heating at 300 °C for 3 hours. On the other hand, direct heating JU101 at 300 °C for 30 minutes (denoted JU101-H) also results in the colour change from colourless to blue, demonstrating a thermochromic effect (Fig. 2a). To the best of our knowledge, the thermally induced electron transfer system, especially in the crystalline solid state, has rarely been reported because high temperature facilitates the charge-recombination. UV/Vis spectra and EPR measurement of JU101-H show the characteristic signals of MV˙^+^ radicals, indicating that thermochromic transformation also arises from the electron transfer process (Fig. 2b and d).

It is worth noting that the photochromic and thermochromic transformation of JU101 is not easily reversible, and the blue colour state is stable in air for at least one month with no obvious change. EPR study shows that the intensity of the EPR signal slightly decreases after JU101-P is kept in the dark for 15 days (Fig. S10, ESI†). Such phenomenon has been observed in two reported MV-based MOFs in which the colour-changed samples can be stable in air for at least two weeks.^19,20^ This implies that JU101 possesses an ultralong-lived charge-separated state upon photo irradiation or heating. The reason may be attributed to the dense packing mode of MV^2+^ cations in the pores of JU101, which prevents the contact of the generated radicals with framework oxygen.^17^ Because long-lived charge separation is crucial for converting solar energy to chemical energy,^34^ JU101 might function as a potential solar energy conversion material.

### Thermal stability

**Fig. 3:**
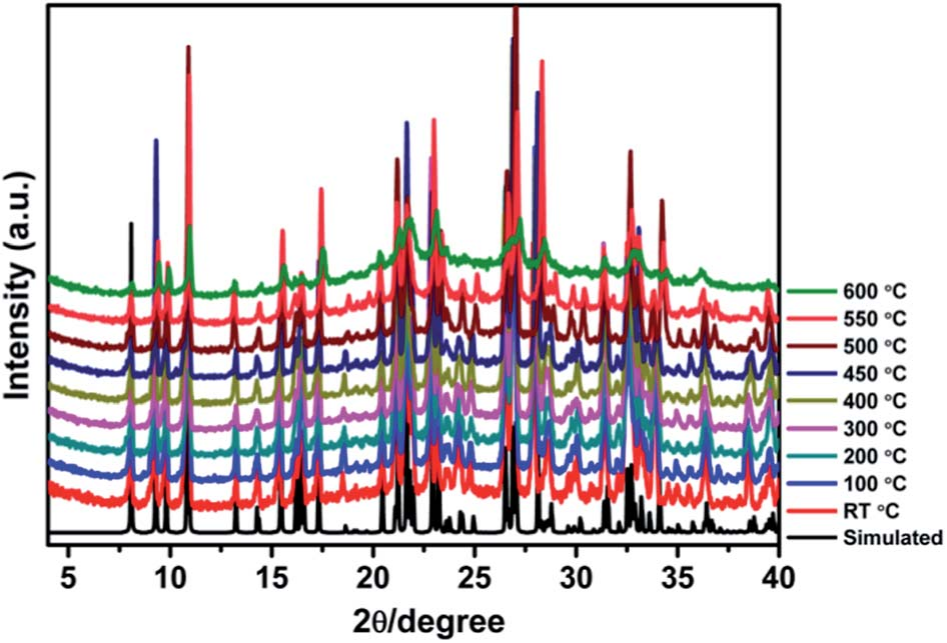
Temperature dependent PXRD patterns of JU101 from room temperature to 600 °C.

High thermal stability is required for the practical applications of crystalline photo-/thermochromic materials. Especially, in the solar energy conversion system, continuous light irradiation with high energy photons concentrated on the crystal surface can cause crystal damage. Temperature dependent XRD analysis indicates that the framework of JU101 can be stable up to 600 °C (Fig. 3). As far as we know, JU101 is the most stable viologen-based photo-/thermochromic material.

### Photovoltaic activity

**Fig. 4:**
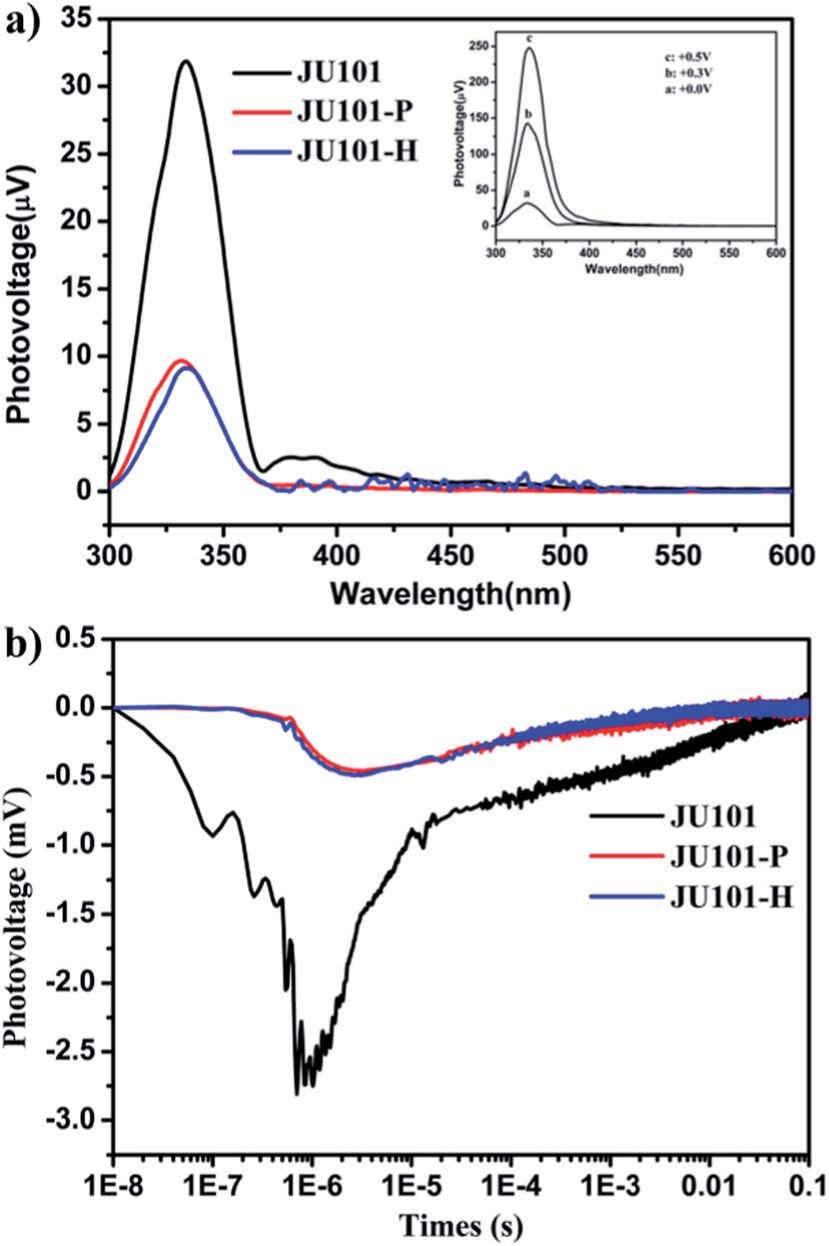
(a) SPV spectra of JU101, photo irradiated sample JU101-P and heated sample JU101-H in the absence of an external electric field. Inset: FISPS of JU101 under different positive electric-fields. (b) TPV spectra of JU101, JU101-P, and JU101-H.

Strikingly, studies by steady state surface photovoltage spectroscopy (SPV) reveal that JU101 also possesses photovoltaic properties. When illuminated without external electric field, an SPV response in the range of 300–370 nm (max. 334 nm) is observed for JU101 (Fig. 4a). Field-induced surface photovoltage spectroscopy (FISPS) study shows that the intensity of the SPV response increases with the enhanced positive electric-field strength, suggesting that the photoinduced charges can be effectively separated by an added positive electrical field. The intensity of the SPV response clearly decreases for JU101 after light irradiation or heating, indicating a tuneable photovoltaic activity in response to light and heating. Transient state surface photovoltage (TPV) response gives a negative signal for JU101 (Fig. 4b), suggesting a semiconductor character. In accord with the SPV response, the intensity and lifetime of the TPV signal decrease after light irradiation and heating. As is known, the photovoltaic effect is based on the photogeneration of excess carriers, followed by their spatial separation. The above results indicate that such a process might be suppressed by the formation of MV˙^+^ radicals under light irradiation and heating. The detailed mechanism of the intriguing photovoltaic properties of JU101 needs further investigation.

## Conclusions

In summary, a multifunctional photochromic and thermochromic zeolitic material JU101 has been synthesized with *in situ* generated MV as the SDA. JU101 exhibits a novel zeolite topology with an interconnecting 8 × 10 × 10 channel system. JU101 presents advantages as a photochromism and thermochromism material: dual photo-/thermoactivated coloration, extended photo-responsive range from UV to visible light, high thermal stability, and ultralong-lived charge-separated state. It also displays tuneable photovoltaic activity coupled with the photo-/thermochromism. The successful synthesis of JU101 and finding of its unusual properties associated with the MV species will open a new vista in searching for materials with useful photophysical functionalities.
