# Supplementary material for: Methyl viologen-templated zinc gallophosphate zeolitic material with dual photo-/thermochromism and tuneable photovoltaic activity
Source: Chem Sci. 2015 Mar 5;6(5):2922–7. doi: 10.1039/c5sc00291e (PMC5490056; doi:10.1039/c5sc00291e)
Supplement: Supplementary file 1 [file SC-006-C5SC00291E-s001.pdf]

Electronic Supplementary Material (ESI) for Chemical Science.
This journal is © The Royal Society of Chemistry 2015

Supporting Information for

Methylviologen-Templated Zinc Gallophosphate Zeolitic Material with Dual Photo-/Thermochromism and Tuneable Photovoltaic Activity

Junbiao Wu, Chunyao Tao, Yi Li, Jiyang Li, Jihong Yu**

State Key Laboratory of Inorganic Synthesis and Preparative Chemistry, College of Chemistry, Jilin University, Changchun 130012 (P. R. China)

E-mail: lijiyang@jlu.edu.cn; jihong@jlu.edu.cn

Table S1. Crystal data and structure refinement for JU101 [a]

Empirical formula	$Ga_{1.67}$ O_{10} $P_{2.50}$ $Zn_{0.83}$
Formula weight	408.10
Temperature	296(2) K
Wavelength(Å)	0.71073
Crystal system, space group	Monoclinic, *C*2/m
Unit cell dimensions	
a (Å)	16.767(3)
b (Å)	19.047(4)
c (Å)	10.248(2)
α(deg)	90
β(deg)	127.038(3)
γ (deg)	90
Volume(Å^3)	2612.4(9)
Z, calculated density(mg m^{-3})	8, 2.075
Absorption coefficient(mm^{-1})	5.277
F(000)	1553
Crystal size(mm^3)	0.22 x 0.21 x 0.18
θ range(°) for data collection	1.860–28.284
Limiting indices	$-22 \le h \le 19$, $-25 \le k \le 25$, $-7 \le l \le 13$
Reflections collected/unique	9591/3334, [R(int) = 0.0442]
Completeness to θ (%)	25.242, 100
Absorption correction	semi-empirical from equivalents
Refinement method	full-matrix least-squares on F^2
Data/restraints/parameters	3334 / 1 / 144
Goodness-of-fit on F^2	1.050
Final *R* indices [$I > 2\sigma(I)$]	$R_1 = 0.0466$, $wR_2 = 0.1257$
R indices (all data)	$R_1 = 0.0677$, $wR_2 = 0.1353$
Largest diff. peak and hole (eÅ^{-3})	1.552 and -0.774

[a] $R_1 = \sum(\Delta F/\sum(F_o))$, $wR_2 = (\sum[w(F_o^2 - F_c^2)])/\sum[w(F_o^2)^2]^{1/2}$ *and* $w=1/[s^2(F_o^2)+(0.0567P)^2+10.3802P]$ *where* $P=(F_o^2+2F_c^2)/3$

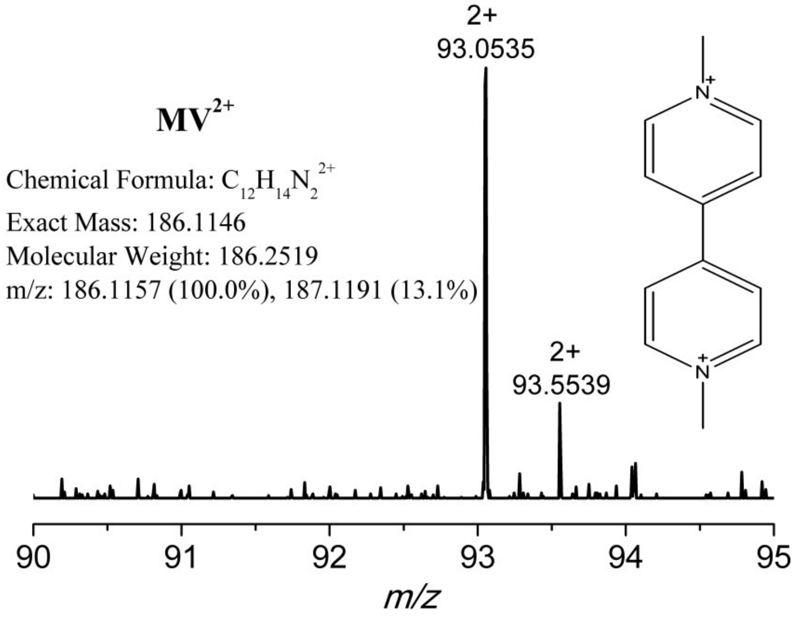

Figure S1. Liquid chromatography-high resolution mass spectrum (LC-HRMS) of JU101.

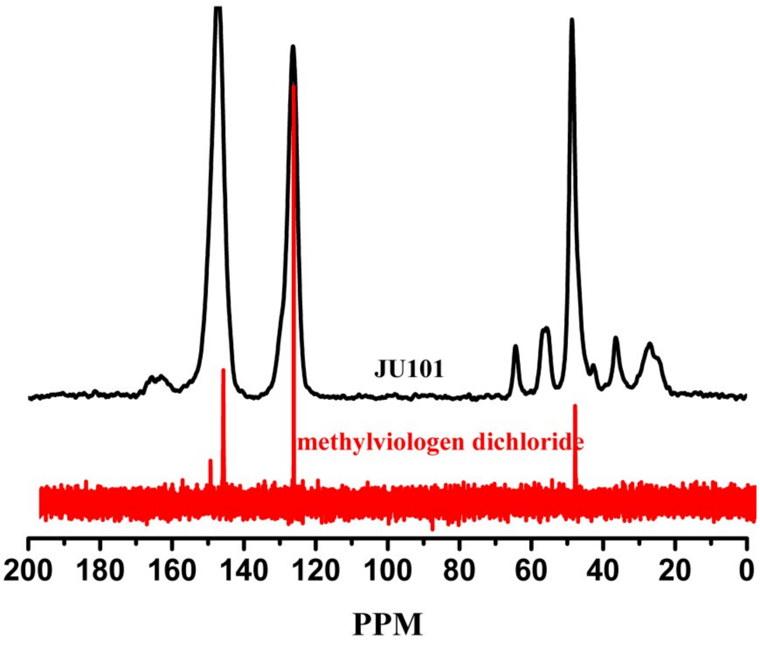

Figure S2. Solid-state ^{13}C MAS NMR spectrum of JU101 and liquid ^{13}C NMR of methylviologen dichloride. Their well agreement indicates that the intact MV^{2+} cations are occluded in the structure of JU101.

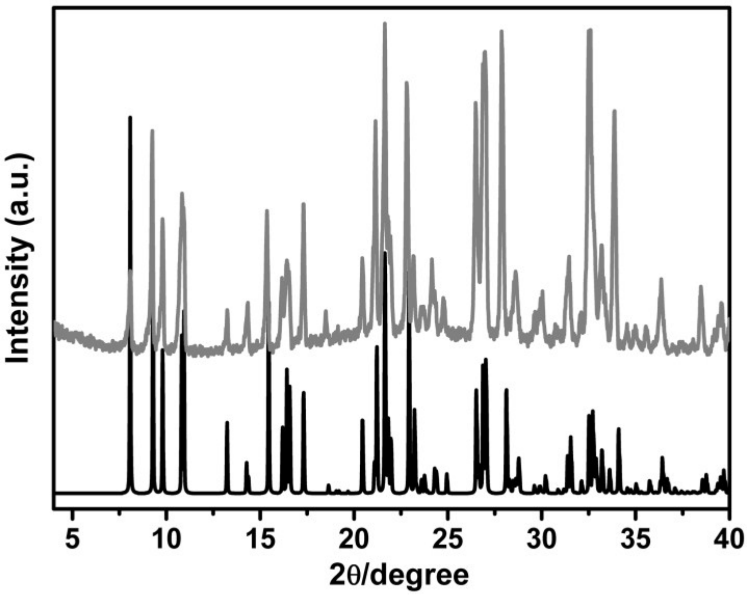

Figure S3. Experimental (top) and simulated (down) PXRD patterns of JU101. Their well agreement suggests the phase purity of the selected crystals.

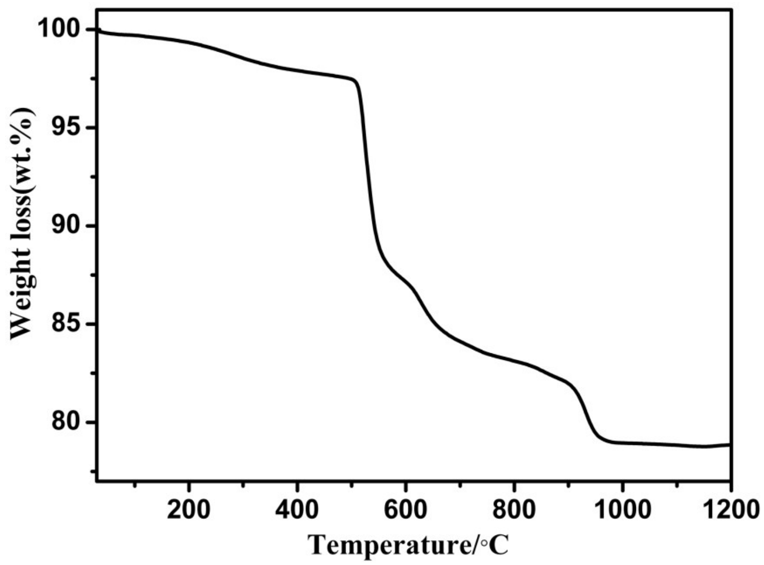

Figure S4. TG curve of JU101 from room temperature to 1200 °C.

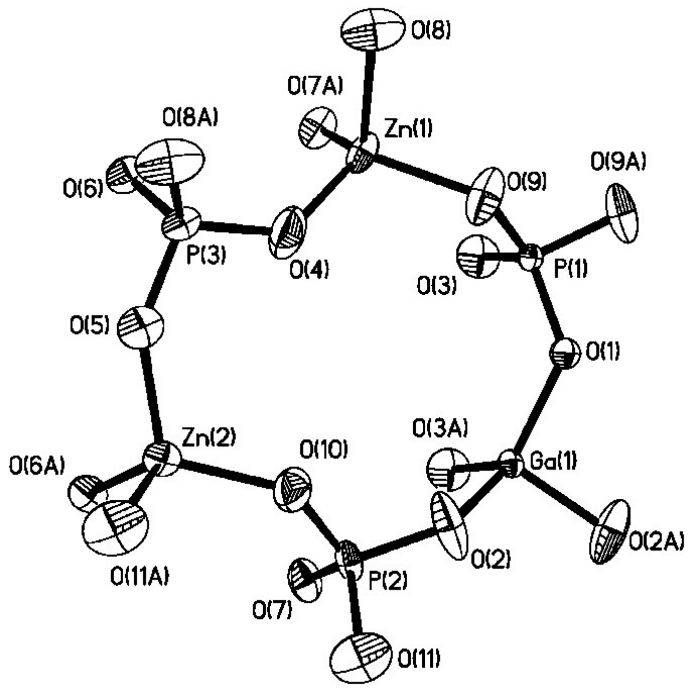

Figure S5. Thermal ellipsoids of JU101 given at 50% probability, showing the atomic labeling scheme (disordered MV^{2+} cations and F^- ions are not given).

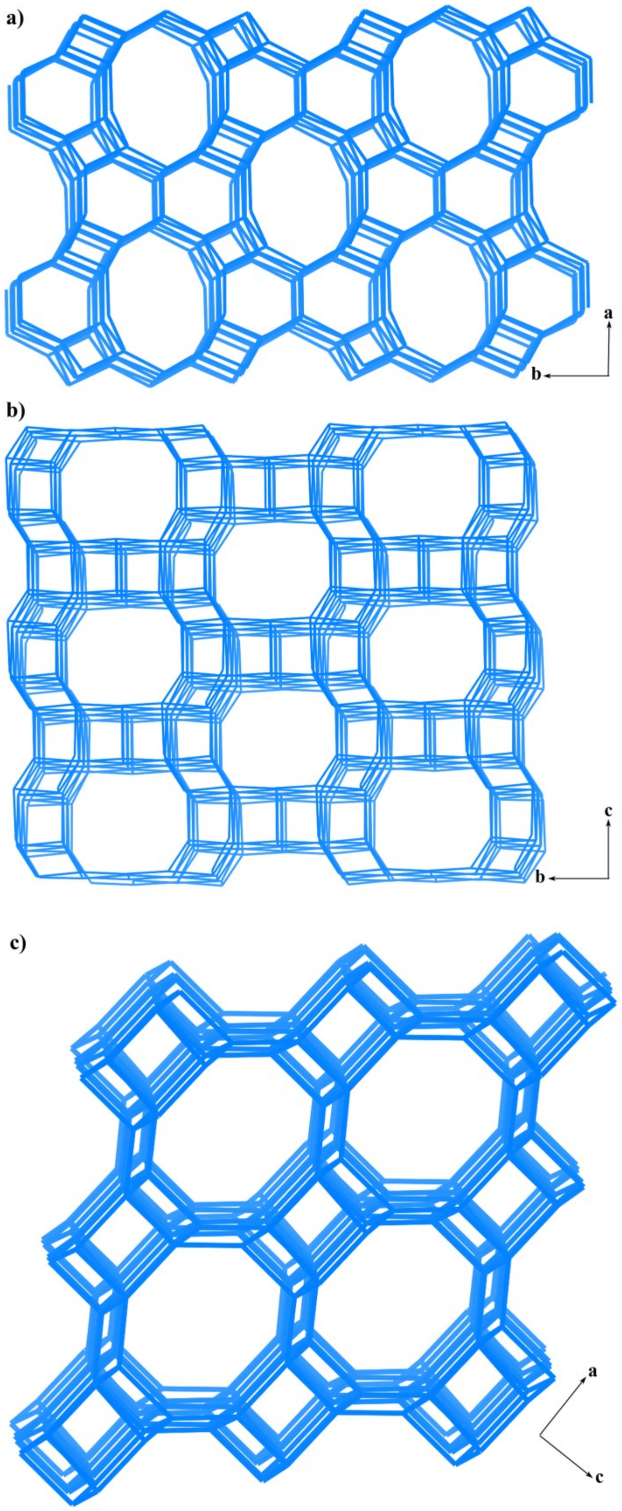

Figure S6. 3D channel system of JU101 composed of 10-ring channels running along the (a) [001] (6.4 × 5.0 Å^2) and (b) [100] (4.5 × 3.6 Å^2) directions, and (c) 8-ring channels running along the [010] directions (4.0 × 3.9 Å^2).

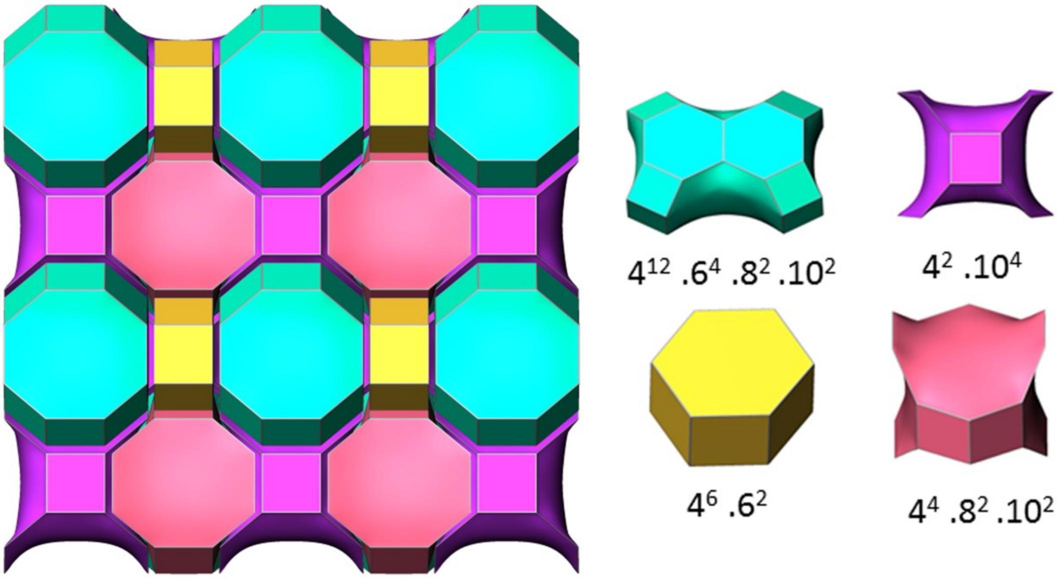

Figure S7. Tiling structure of JU101 viewed along the [001] directions. Four types of tiles are shown in different colors.

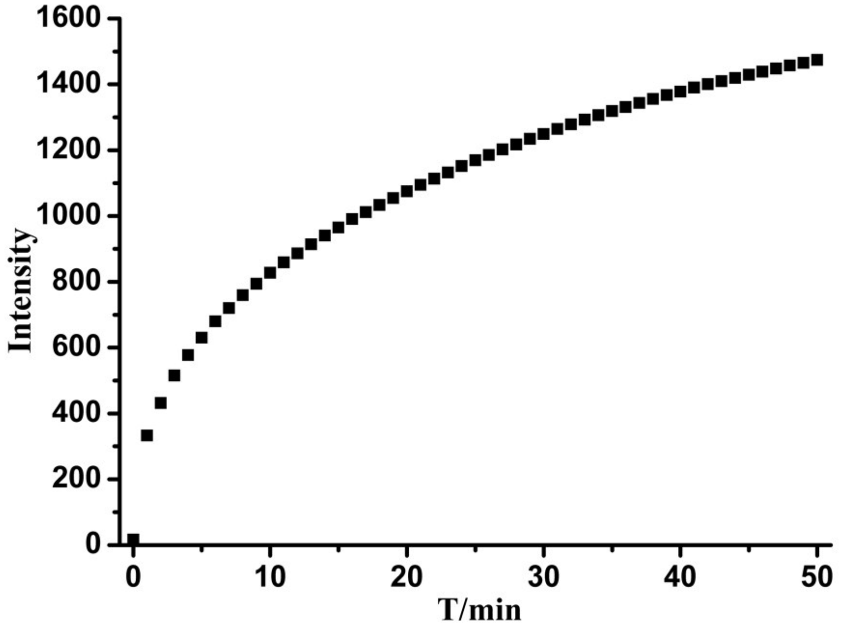

Figure S8. Intensity of EPR signals of JU101-P with prolonged UV irradiation time.

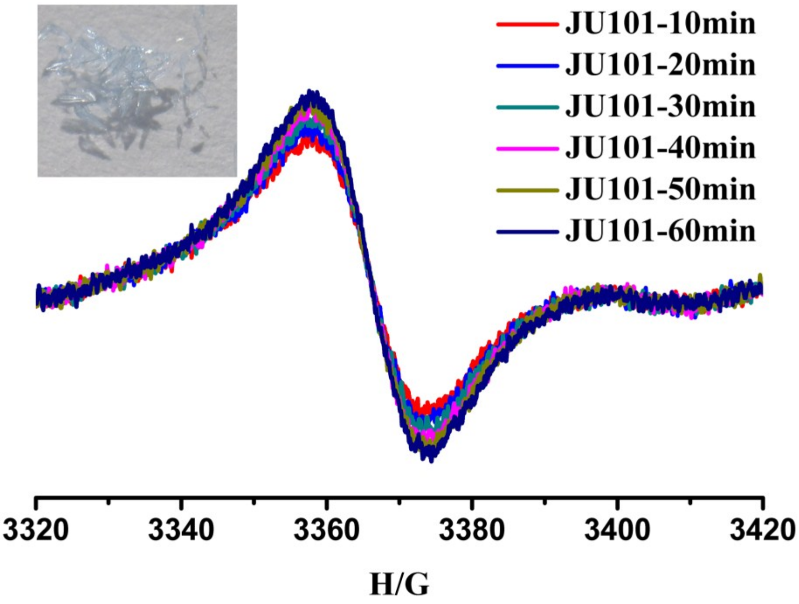

Figure S9. *In-situ* time dependent EPR spectra of JU101 under visible light illumination (insert is the photograph of JU101 illuminated upon visible light for 1h).

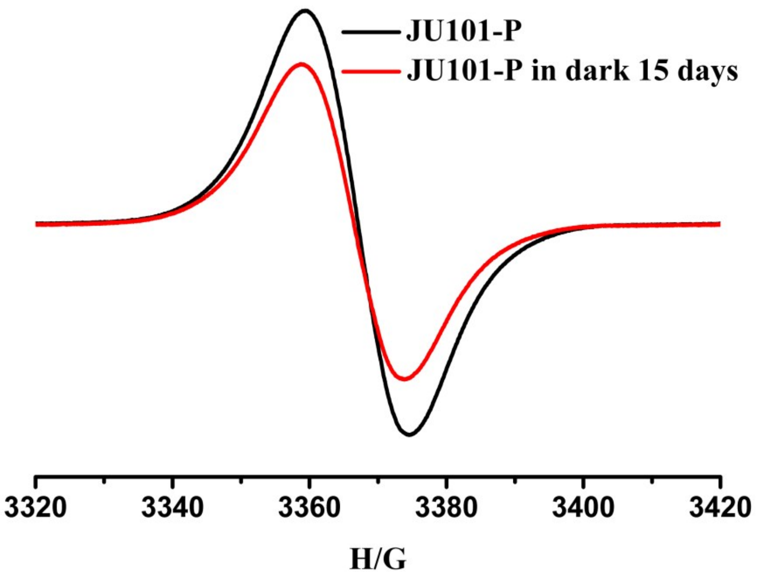

Figure S10. EPR signals of JU101-P and JU101-P kept in the dark for 15 days.
